# The influence of participation in work‐related activities on mental well‐being among people with dementia or mild cognitive impairment: A multicenter cross‐sectional study in Japan

**DOI:** 10.1002/alz70858_099629

**Published:** 2025-12-25

**Authors:** Erika Kamo, Yuma Sonoda, Takuma Yuri, Kayano Yotsumoto, Hisatomo Kowa

**Affiliations:** ^1^ Kobe University Graduate School of Health Sciences, Kobe, Hyogo, Japan; ^2^ Kobe University Advanced Research Center for Well‐being, Kobe, Hyogo, Japan; ^3^ Kyoto Tachibana University, Kyoto, Kyoto, Japan; ^4^ School of Health Sciences, Kobe University, Hyogo, Japan

## Abstract

**Background:**

People with dementia or mild cognitive impairment (MCI) often experience unmet needs and prescribed disengagement in meaningful activities. Social participation through work‐related activities has the potential to improve the mental well‐being of people with dementia or MCI. However, there is a shortage of research on the effects of work‐related activities on the mental well‐being of people with dementia or MCI. This study aimed to investigate the influence of participation in work‐related activities on mental well‐being and its associated factors in people with dementia or MCI.

**Method:**

The study design was a multicenter cross‐sectional study. We administered questionnaires and measurements to participants who were members of a day service and had dementia or MCI. We collected the World Health Organization‐Five Well‐Being Index (WHO‐5) as the main outcome and its confounders, such as age, sex, educational history, economic status, hospitalization history, dementia severity, cognitive function, vision, hearing, physical pain, and physical and social frailty. These data were compared between the work‐related activities and the usual care groups. Multiple regression analysis was performed with WHO‐5 as the dependent variable and participation in work‐related activities and WHO‐5 covariates as independent variables.

**Result:**

A total of 128 participants were compared between the two groups. The work‐related activity group had better mental well‐being measured by WHO‐5 than the usual care group (median [Interquartile Range]: 18.00 [11.00, 25.00] vs. 15.00 [8.00, 24.00], *p* < 0.001). The same results of WHO‐5 were obtained about after adjusting for covariates of participant using propensity score matching (median [Interquartile Range]: 18.00 [11.00, 25.00] vs. 14.50 [8.00, 24.00], *p* < 0.001). Multiple regression analysis identified participation in work‐related activities (95% Confidence Interval: ‐4.792, ‐1.556, *p* < 0.001) and social frailty (95% Confidence Interval: ‐3.369, 0.020, *p* = 0.056) as factors that potentially influence WHO‐5. The adjusted R‐squared for this model was 0.26 (*p* < 0.001).

**Conclusion:**

Participation in work‐related activities positively influenced the mental well‐being of people with dementia or MCI. Longitudinal studies are required to investigate the effects of work‐related activities on the mental well‐being of people with dementia or MCI.

